# High-Resolution Insights Into the *in vitro* Developing Blood-Brain Barrier: Novel Morphological Features of Endothelial Nanotube Function

**DOI:** 10.3389/fnana.2021.661065

**Published:** 2021-06-25

**Authors:** Shireen Mentor, David Fisher

**Affiliations:** ^1^Neurobiology Research Group, Department of Medical Biosciences, Faculty of Natural Sciences, University of the Western Cape, Cape Town, South Africa; ^2^Adjunct Professor in School of Health Professions, University of Missouri, Columbia, MO, United States

**Keywords:** blood-brain barrier, exosomes, nanovesicles, tunneling nanotubes, tethering nanotubes

## Abstract

High-resolution electron microscopy (HREM) imaging of the *in vitro* blood-brain barrier (BBB), is a promising modality for investigating the dynamic morphological interplay underpinning BBB development. The successful establishment of BBB integrity is grounded in the brain endothelial cells (BEC’s) ability to occlude its paracellular spaces of brain capillaries through the expression of the intercellular tight junction (TJ) proteins. The impermeability of these paracellular spaces are crucial in the regulation of transcellular transport systems to achieve homeostasis of the central nervous system. To-date research describing morphologically, the dynamics by which TJ interaction is orchestrated to successfully construct a specialized barrier remains undescribed. In this study, the application of HREM illuminates the novel, dynamic and highly restrictive BEC paracellular pathway which is founded based on lateral membrane alignment which is the functional imperative for the mechanical juxtapositioning of TJ zones that underpin molecular bonding and sealing of the paracellular space. For the first time, we report on the secretion of a basement membrane *in vitro*, which allow BECs to orientate themselves into distinct basolateral and apicolateral domains and establish a 3-dimensional BEC construct. We report for the first time, on the expression of nanovesicles bound to the plasma membrane surfaces of the BECs. These membrane-bound vesicles are reported to possess an array of DNA/RNA constituents and chemotaxic properties affecting the formation of nanotubes that span the paracellular space between BECs, facilitating BBB construction, alluding to a functional role in mediating cell-to-cell communication. This study suggests that novel, ultrathin nanotubular (NT) structures are involved in functional roles in bringing into alignment the paracellular space of BECs. Immortalized mouse BECs (b.End3, b.End5) and primary rat cardiac microvascular ECs were used to further validate the *in vitro* BBB model by profiling variances in peripheral EC monolayer development. These cardiac capillary ECs presented with an opposite topographical profile: large fenestra and intercellular spaces, devoid of morphological ultrastructures. This comparative study alludes to the role of NT facilitation in TJ-induced hemifusion of apicolateral BEC membranes, as a structural event forming the basis for establishing a polarized BBB.

## Introduction

Endothelial cells (ECs) originate from the mesoderm, a germ layer that forms at gastrulation, during early embryonic development (Dyer and Patterson, 2010) and are essential for capillary formation. Vasculogenesis involves the formation of major vessels in the embryonic midline from angioblasts that originate in the lateral plate mesoderm (Risau and Flamme, 1995; Okuda and Hogan, 2020). The vascularization of the brain and spinal cord begins before birth, by way of angiogenic sprouting networks, namely the perineural vascular plexus (PNVP) and the periventricular plexus (PVP) (Ruhrberg and Bautch, 2013). The PNVP arises from the mesoderm-derived angioblasts (endothelial precursor cells) and conceals the entire central nervous system (CNS) by embryonic day 9.0 (E9.0) (Engelhardt and Liebner, 2014; Gupta et al., 2021). Brain endothelial cells (BECs) grow in close proximity forming restrictive capillary tubes due to the presence of barrier points. The literature denotes these contact points as “kissing points” which is further supported by freeze-fracture studies (Haseloff et al., 2015). In the current morphological study it is referred to as “stitching points.” Based on the literature, this partial “stitching” of the BEC membrane appears to be a central part of blood-brain barrier (BBB) CNS vascularization. The development of the BBB is dependent on BECs aligning themselves along their lateral membranes in such way that an array of transmembrane tight-junction (TJ) molecules from adjacent BECs can physically connect, very specifically apicolaterally, sealing the paracellular (PC) space. Although the TJs are fundamental to the integrity of BBB, the mechanisms involved in the alignment between two adjacent cells, has not been described in the literature.

In the current study, the key focus is on how the BBB is forged, primarily, by the BECs of the cerebromicrovasculature interconnected by intercellular TJ protein complexes (Saunders et al., 2014; Qosa et al., 2016). BECs are key components to BBB integrity and regulates the homeostatic *milieu* of the brains microenvironment, by the strict control of the permeability of its capillaries. The BBB regulatory mechanisms, despite its overall strength, is a persistent impediment in the successful treatment of CNS associated diseases (i.e., Alzheimer’s Disease, Parkinson’s Disease, Brain Cancer etc., actively precluding the entry of drugs to target areas within the diseased brain).

Previously, imaging the molecular interplay between BBB-ECs was encumbered by methodological difficulty. We, hereby, utilize an innovative experimental design to visualize the molecular architecture of brain capillary endothelium development, and with the utilization of high-resolution scanning electron microscopy (HRSEM), the visualization of *in vitro* BBB morphogenesis into a functional monolayer has dramatically enlightened our understanding with regards to how morphological ultrastructures orchestrate adjacent BEC alignment to facilitate the molecular interaction between TJ proteins.

Intercellular communication between BECs is essential to accomplish molecular alignment and the proliferating environment of the brain capillary EC is highly dependent on paracrine signaling molecules, which includes an array of growth factors, e.g., tissue necrotic factor beta, and vascular endothelial growth factor etc. (Lucas et al., 2009). In angiogenesis, cell-to-cell communication is achieved by way of paracrine, autocrine, endocrine factors and by direct cell-to-cell contact, but how this contributes to aligning cells to accomplish the molecular connection between corresponding PC rows of TJs within the cerebromicrovasculature, was until now, still largely unknown.

*In vitro* models of the BBB have long been used to elucidate the physiological functioning of the *in vivo* BBB, as well as the mechanisms involved in various experimental and clinical treatment procedures. However, except for a few studies, information on the development of the formation of the functional *in vitro* monolayer has remained theoretical at best. Based on our HRSEM studies on the immortalized mouse brain endothelial cells (b.End3 and b.End5), we introduce a novel technique to report, how nanovesicles (NV) induced nanotubular (NT) structures (Gerdes et al., 2007; Gurke et al., 2008; Gerdes et al., 2013) extending from the plasma membrane, can contribute to the alignment of apical membrane proteins during BBB development. To date, there are a plethora of terms used to coin the varying sizes of extracellular vesicles exocytosed from cells (Tamkovich et al., 2016; Schwich and Rebmann, 2018). It is thus propitious to bring clarity to the manner in which we denote these extracellular vesicles (EVs). Different sources use different terms (viz. microparticles, microvessesls, ectosomes, shedding microvesicles, NV, exosomes, exosome-like particles, dexosomes, texosomes, and oncosomes etc.) each term employed according to the various biological material in which they reside [(7) Tamkovich et al., 2016] (Table 1).

**TABLE 1:** The four major categories of extracellular vesicles.

Vesicle	Mechanism of generation	Size (nm-μm)	References
1. Exosomes	Microvesicular endocytic process; endosomal membrane particle	30–100 nm 70–150 nm	Tamkovich et al., 2016; Schwich and Rebmann, 2018
2. Nano vesicles (NV)	Exocytosed extracellular vesicles	30–300 nm	Novel
3. Microvesicles (μm)	Outward budding and scission of plasma membrane	50–500 nm 100 nm-1,000 μm	Tamkovich et al., 2016; Schwich and Rebmann, 2018
4. Oncosomes and blebbing/apoptotic bodies	Generated from apoptotic bodies and amoeboid cancerous cells	>500 nm	Schwich and Rebmann, 2018

NTs can be further classified into tethering NTs (TENTs) and tunneling NTs (TUNTs), which are crucial to the intercellular interplay which mobilizes the molecular alignment of TJs between adjacent BEC; requisite to the mechanical juxtapositioning of the BECs and its alignment into a physiological BBB which begins with qualitative and quantitative assessment of the biophysical properties of BECs during development and proliferation, enabling the step-by-step analysis of the evolution of a highly restrictive, continuous membranous structure.

## Materials and Methods

### Bio-Reagents

The immortalized mouse brain endothelial cells (b.End3) were pre-incubated in standard Dulbecco’s Modified Eagles Medium: Hams F12 DMEM: F12 nutrient mixture (Thermo Fisher, Cat no. 2176317) supplemented with 1% *Penicillin-Streptomycin* (Whitehead Scientific (Pty) Ltd., Cat no. 17-745E) and 0.117 g of L-glutamine (4 mM) (Sigma-Aldrich, Cat no. G-3126) and b.End5 cells were grown in DMEM: F12 (BioWhitakker/Lonza^®^, Cat no.12-719F), supplemented with 1% *Pen/Strep*, 1% non-essential amino acids (BioWhitakker/Lonza^®^, Cat no.13-114E), 1% Sodium pyruvate (Gibco^®^, Cat no. 11360) and 10% Fetal bovine serum (Celtic Diagnostics/BioWest, Cat no. S181G-500).

### Tissue Culture

#### Immortalized Mouse Brain Endothelial Cell

Both brain endothelial cell lines were derived from BECs of BALB/c mice (Watanabe et al., 2013). The only difference designated to each cell line is the company from which each was purchased. The b.End3 was purchased from American Type Culture Collection, Cat no. CRL-2299 and are positive for gene expression of: von Willebrand factor, Intercellular Adhesion Molecule 1; Vascular cell adhesion protein 1 (VCAM-1) and the mucosal vascular addressin (MAdCAM-1). The MAdCAM-1 and CD62 antigen-like family member E. The b.End5 cell lines was purchased from the European Collection of Authenticated Cell Cultures, Cat no. 96091930 and are positive for gene expression of endothelial specific proteins: Platelet endothelial cell adhesion molecule, Endoglin, Panendothelial Cell Antigen Antibody (MECA-32) and a receptor for vascular endothelial growth factor (Flk-1) tested by fluorescence activated cell sorting. Inflammatory cytokines are able to induce the expression of proteins such as: VCAM-1 and E-selectin.

#### Primary Rat Cardiac Microvascular Endothelial Cell

The primary rat cardiac microvascular endothelial cell (CMEC) line is a primary line, was donated by Dr. A. Genis, at Stellenbosch University-Tygerberg campus, Tygerberg, Cape Town, South Africa.

##### CMEC Cell Culture

The CMECs are seeded on TC plates that are pre-coated for an hour with attachment factor (basically gelatin), from Life Technologies. The confluent plates are trypsinized with 0.25% Trypsin-EDTA solution (Whitehead Scientific, Cat no. BE 02-007E) and suspended cells are removed and re-plated in a 1:2 ratio. Suspended cells are centrifuged at 1000 rpm for 3 min to obtain a pellet. The pellet is re-suspended in a specialized growth medium. Microvascular Endothelial Cell Growth Medium-2 (Whitehead Scientific-Lonza^®^, Cat no. CC-3156) and supplemented with the Bullet Kit (Whitehead Scientific-Lonza^®^, Cat no. CC-4147), when ordered together as one package, the Cat no. is CC-3202.

### The *in vitro* Bicameral System

Immortalized mouse BECs and primary rat CMECs were grown on an insert membrane with a 12 mm diameter, a pore size of 0.45μm and an effective filtration area of 0.6 cm^2^. The membrane of the insert was comprised of mixed cellulose esters, according to the manufacturers’ specifications (Millicell^®^ insert, (Merck), Cat no. PIHA01250). Inserts were placed in 24-well tissue culture microtiter plates (Adcock Ingram). BEC monolayers (b.End5) were seeded on the inserts at a low cell density (1 × 10^4^ cells/insert/well), to allow for sparse location of cells, and both CMECs and BECs were seeded at cell densities ranging from 1 × 10^4^ −1 × 10^6^ cells/insert/well, respectively, so that the close proximity of cells could facilitate monolayer confluence, and also to promote cell-to-cell communication over a 24–48 h timeframe.

To identify PC structures, we designed a cell culture experiment that provided the BEC with a slightly hypertonic tissue culture environment (330–340 mosmol/kg) to promote subtle crenation of the BEC body. The slight crenation allowed for the PC space to be uncovered which better permits TUNT and TENT ultrastructural investigation along the adjacent, lateral cell membranes.

### Scanning Electron Microscopy

We introduce a novel technique that involves growing BECs to confluence on inserts and, thereafter, image monolayer development utilizing HRSEM. BECs (b.End5 cells) were grown at 37°C, at 5% CO_2_ on Millicell filter inserts (at 1 × 10^4^ and 1 × 10^6^ cells/insert/well). Upon cellular confluence, cell monolayers were fixed with 2.5% glutaraldehyde solution (BioChemika/Fluka- Sigma-Aldrich) (Faso et al., 1994). The biological sample was dehydrated in a graded series of ethanol concentrations and critically dried using a Hitachi HCP-2 critical point dryer. Samples were coated with gold-palladium (Au: Pd) and imaged using a Zeiss Auriga high-resolution field-emission gun SEM. All images were captured using an in-lens secondary electron detector.

### Transmission Electron Microscopy

The b.End3 cells were grown at 5 × 10^5^ cells/insert/well at 37°C and 5% CO_2_ (*n* = 3; day = 0) in supplemented DMEM: F12. Cells were allowed to attach and expand to confluence for 24 h. The samples were chemically fixed in 2.5% glutaraldehyde solution (BioChemika/Fluka- Sigma-Aldrich), in 100 mM phosphate buffer at pH 7.2) for 2–24 h at 4°C (Faso et al., 1994). Samples were washed twice in 100 mM phosphate buffer that has been adjusted to the osmolarity of the sample to prevent tissue damage. Post-fixation was conducted using 1% Osmium tetroxide made in 100 mM phosphate buffer 1–2 h, at 4°C. Specimens were incubated in 2% aqueous uranyl acetate and dehydrated in a graded series of ethanol concentrations. Samples were embedded using a series of resin-ethanol mixes during the infiltration process. Ultrathin thin sections (∼60 nm) slices of sample embedded in resin were prepared using a Reichert Ultracut S ultramicrotome. Sections were imaged on a FEI/TECNAI T20 transmission electron microscope.

## Results

This study strongly suggests how novel, nanosized ultrastructures functionally cooperate in the formation of a physiologically functional *in vitro* BBB model. Furthermore, we discuss novel mechanisms showing how tunneling and tethering nanotubules (TUNTs and TENTs) may play an important role in aligning the brain capillary endothelial cells to form sealed PC pathways. This study describes how BECs develop morphologically in an *in vitro* environment designed to model the BBB using HRSEM to morphological describe cellular membrane ultrastructures on a nanoscale. This is especially pertinent to a more informed perspective of the *in vivo* formation of the BBB.

### *In vitro* Secretion of the Basement Membrane

**FIGURE 1:**
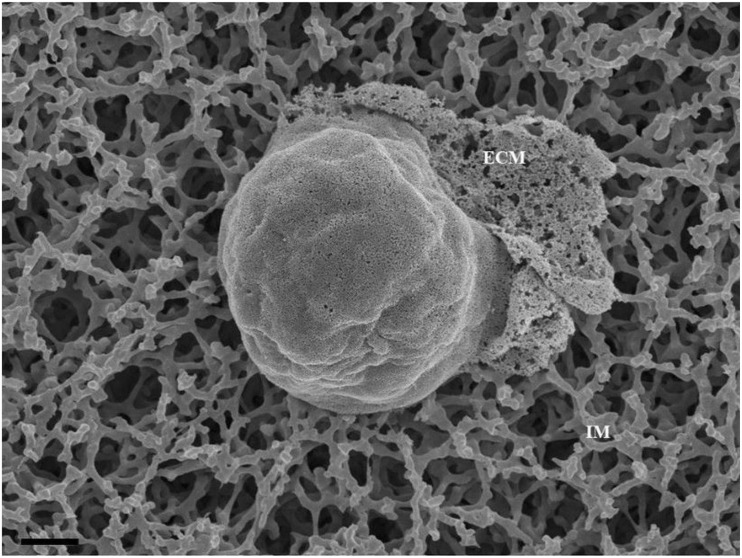
An HRSEM micrographic illustration of a b.End5 cell seeded on a mixed cellulose insert membrane establishing 3-dimensional cellular architecture (Scale bar = 2000 nm). The micrograph exhibits the secretion of a basement like extracellular matrix (ECM). The insert membrane is denoted as “IM.”

BECs were grown at a low cell density (1 × 10^4^ cells/insert/well) to track its development progressively over 24 and 48 h. At these low cell densities, the BECs were sparsely located, allowing for the investigation of three-dimensional development of cells before exponential cell division resulted in the close approximation of cells in culture and their interaction (Figure 1). The mixed cellulose ester insert membrane mimics a biological surface which allowed cells to orientate themselves and to differentiate functionally into distinct morphological apical and basolateral domains. After the attachment of BECs to the insert membrane the secretion of an amorphous extracellular material from the basal surface of the cell was observed (Figure 1). The HRSEM enabled the viewing of the cell membrane, in which pore-like structures could be identified (Figure 1).

### Nanovesicles

Following repetitive divisions of cells on the insert membrane, it became apparent that the surface or membrane morphology of the cells became increasingly more complex. Two structures became abundant on the cell surface: copious amounts of NVs and the subsequent emergence of primordial, nano-sized filaments emanating from the extracellular apicolateral membrane surfaces.

There are two types of NVs, which we categorized according to possible functions:

#### Chemotaxic Nanovesicles

**FIGURE 2:**
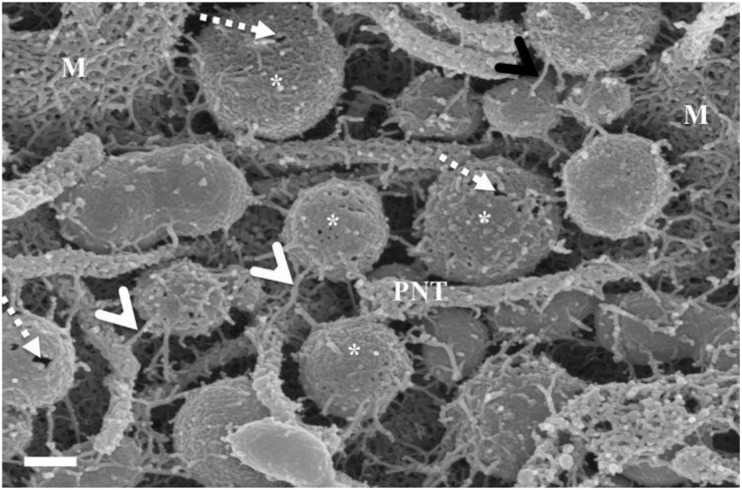
The HRSEM micrograph depicts nanovesicular structures emanating from the b.End5 cell (Scale bar = 200 nm). This category of EV is porous in nature enabling them to act as generators of a chemotaxic gradient and also protrude sticky filaments (white arrowheads), further enabling it to be anchored close to their area of formation. These EVs range from 30 to 300 nm in size and thus are categorized as nanovesicles. The “perforated white arrows” indicate the pores of the exosomes. “**M**” denotes the plasmalemma outer surface, the * asterisk denotes the nanovesicle (NV) and “**PNT**” denotes primordial NTs.

These NVs have membranes that are “adhesive” and are characterized by prominent membrane pores and “sticky” filamentous surface structures. The images suggest that NVs are able to facilitate additional anchorage onto the parent cell membrane (Figure 2). Their membrane structure appears to be identical to the parent cell’s membrane. Although we have not been able to report on the actual formation of these NVs, within a closed cell culture environment, its origins preclude any other contingency other than the cultured cells. It is also of interest to note that initially, upon the seeding of the BECs, there is an absence of NVs; its pervasion only increases upon the formation of denser cell populations. The formation of the NVs are limited to cells growing in close proximity (Figure 2).

#### Nanovesicle-Induced Nanotubules

A novel finding of this study showed that closely approximated NVs, which have been classified into two sub-populations of membrane-bound vesicles, was integrally involved in regulating PC cell-to-cell communication. This study proposes the following: (i) NVs are porous in nature, possessing “sticky” tentacles which allow for the vesicle to attach to the cell membrane (Figure 2), and (ii) a clearly different category of NVs which fused to each other to form nanotubes (NTs), with their proximal ends firmly attached to the surface membranes of the parent cell, while the distal ends, initially forming close-ended, “sticky” ends on the cell membranes of adjacent BECs (see Figures 4A–C), and later these proximal and distal ends fuse with the plasma membranes, connecting the cytoplasm of adjacent cells (refer to Supplementary Figure 1). These NTs appeared to have hollow lumens which stretched across the PC spaces of adjacent BECs appearing to connect the two cells, forming a framework of tubes across the PC space of adjacent BECs.

**FIGURE 3:**
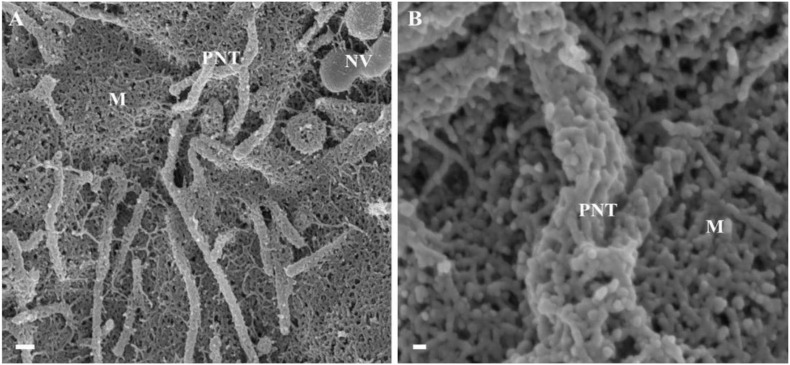
The *de novo* formation of primordial nanotubules on the membrane surface of BECs. **(A)** Depicts a micrograph showing elongated NT structures emanating from the plasmalemma of a b.End5 cell (Scale bar = 200 nm). Membranous protrusions from the cell surface morph into rope-like, tethering NTs on the apical surface of a b.End5 cell membrane. **(B)** Illustrates the formation of dense NT distensions’ originating from the cell membrane surface (Scale bar = 100 nm). “M,” denotes the porous membrane, “NV” denotes the nanovesicle and “PNT,” denotes the primordial nanotubule.

**FIGURE 4:**
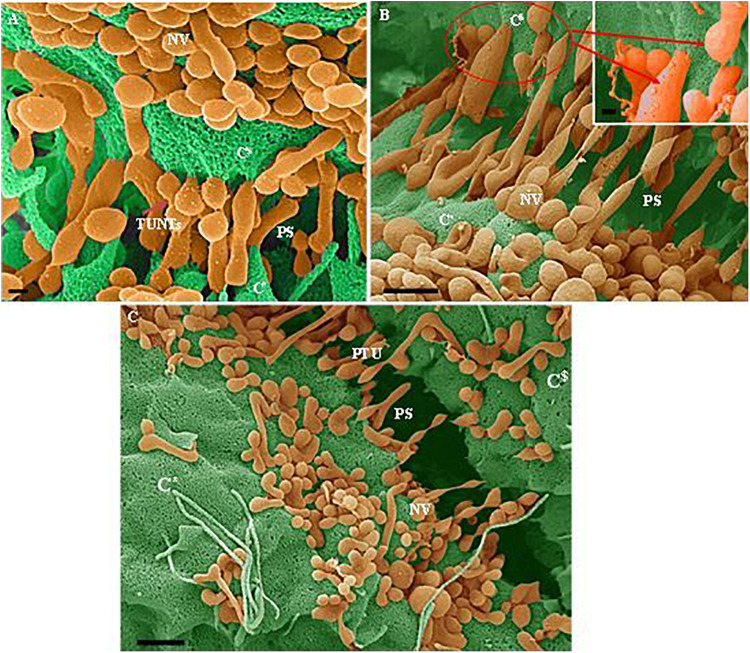
**(A–C)** Indicate both the porosity and the sticky filaments of NVs found along the lateral walls lining the PC space. C* and C^$^ are denoted as cell 1 and cell 2, “**NV**” denotes the nanovesicle, “**PS**” denotes the PC space and “**PTU**” denotes the primordial TUNTs. **(A)** Scale bar = 200 nm; **(B,C)** Scale bar = 1,000 nm.

### Primordial TENTs

HRSEM analysis after 24–48 h of seeding of 1 million b.End5 cells on a cellulose insert membrane showed the *de facto* initiation of projecting cytoplasmic tube-like extensions from the surface of cells (Figures 3A,B). These micrographs illustrate the primordial formation of NT structures extending from the cell membrane surface, eventually developing into NT cross-bridges across the PC space. The outer surface of the primordial NTs appears to be consistent with the molecular structures of the cell membrane (Figure 3B). Sparsely distributed BECs did not express the elaborate membranous structures and are devoid of structures involved in cell-to-cell communication, including exocytotic vesicles and subsequent NT formation (Figure 1), which was only observed when cell populations grew to the close proximity of each other (Figure 3).

In Figures 4A–C NVs are exocytosed onto the BEC PM surface and remain “attached” to the cell membrane. The NVs progress from mono-vesicular structures, by a process of fusion, to form bi-vesicular, tri-vesicular and multi-vesicular structures which adjoin to form elongated, NTs. These NTs morph into “tunneling tubes which have a clear trans-paracellular space lineament as it propagates across the PC space along the apicolateral domain. This NV-induced, tubular formation appears to be the *de novo* synthesis of a tunneling NT (TUNTs) and to the best of our knowledge, it has never been described before in the literature. The TUNT, thus comprises of all amalgamated contents housed within individual NV packages.

**FIGURE 5:**
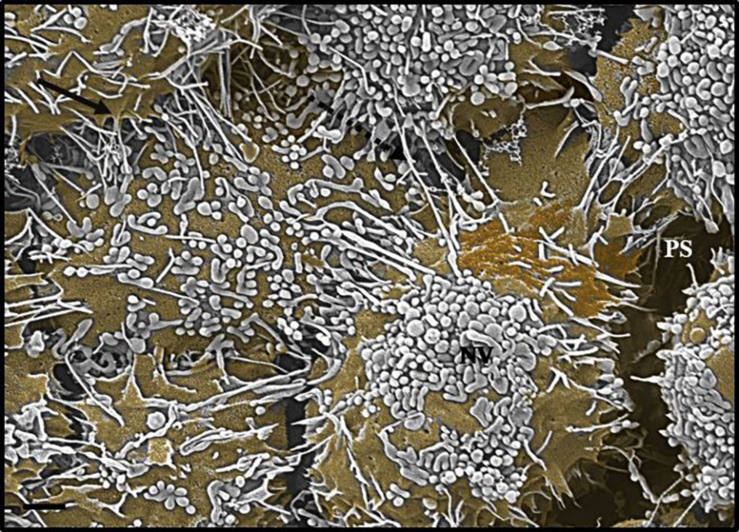
An HRSEM micrograph 24 h after seeding b.End5 (BECs) in close proximity (1 × 10^6^ cells/insert/well) (Scale bar = 1000 nm). The nanovesicle is denoted by an “**NV,**” **TUNTs** are indicated by the perforated, black arrow and **TENT** formation is indicated by the solid, black arrow and the “**PS,**” denotes the PC space.

In Figure 5, we see cells have been purposefully crenated to expose some of the morphological topography between adjacent cells. During the crenation process, some of the NTs which have been attached to adjacent cells have been broken. This process exposed the molecular and morphological membrane processes involved in aligning and joining these PC spaces (Figure 5).

### BEC Tethering Nanotubules

**FIGURE 6:**
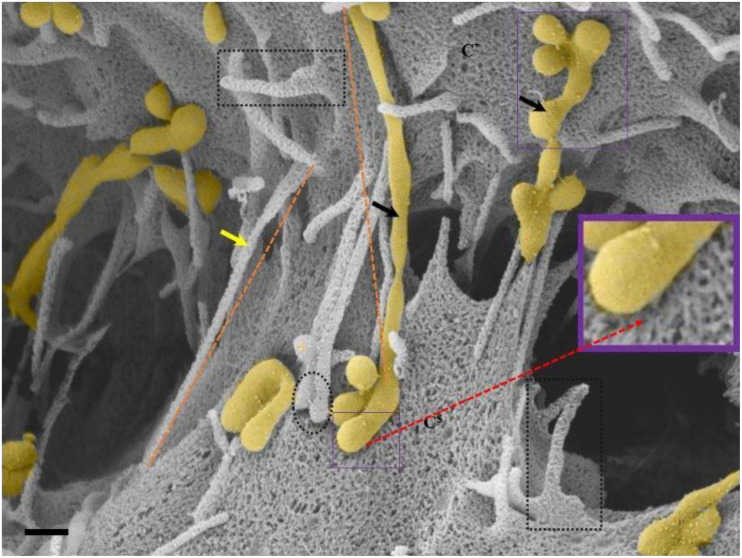
An HRSEM micrograph of two BECs utilizing TENTs to facilitate occlusion of the PC space (Scale bar = 200 nm). Proximal ends of short developing TENTs (**perforated rectangles**) are observed alongside fully extended and developing TUNTs, on each of the juxtaposed lateral membrane surfaces of two BECs in close proximity. TENTs are indicated by the “**yellow arrow,**” its distal ends are indicated by “**black perforated circles**” and TUNTs are indicated by the “**black arrows**.” The proximal and distal ends of TUNTs are indicated by the “**solid purple squares**.” The micrograph depicts the interplay between flanked TENTs and TUNTs which results in the continuity of BEC membrane topography.

Observations of the PC spaces of closely juxtaposed b.End5 cells revealed that NTs are characterized by thin, rope-like structures which functions by mechanically aligning the PC space and subsequently pulling over or securing of plasma membranes across the PC space, playing an important role in the occluding of the PC pathway (Figures 5, 6). Based on the function and the morphological description of the NT we, hereafter, denoted these NTs as tethering NTs (TENTs).

TENTs are characterized by thin filamentous structures (see Figure 6 “yellow arrows,” below), have mechanical integrity and extend with focus intent to project across the PC space and make contact with the adjacent lateral cell membrane its distal ends anchor to the membrane of the adjacent cell.

Proximal ends (origin of the TENT): Scrutiny of the origin of the TENT’s shows that TENTs are continuous and have similar surface cytoarchitecture with the cell’s PM. Furthermore, the proximal origins of TENTs are clearly characterized by triangular membrane leaflets, the apex of which continues to form an NT projecting toward the adjacent cell (see the perforated black frame in Figure 6). Conversely, the proximal end of a TUNT is formed by the fusion of multiple NVs (see Figure 6: purple frame).

Distal ends: the distal end of the TENT is bulb-shaped and appears “sticky” (see the black perforated circle in Figure 6). Initially, close observation of the distal bulb of the TENT shows tiny filaments which appear to anchor the TENT to the lateral membrane of the adjacent cell. With time the distal ends of the TENT appear to be reabsorbed adjacent cell membrane, resulting in the bulb end to disappear. Conversely, the distal end of a TUNT forms foot-like ends which interact with the plasma membranes of the target cell (see purple frame, Figure 6).

**FIGURE 7:**
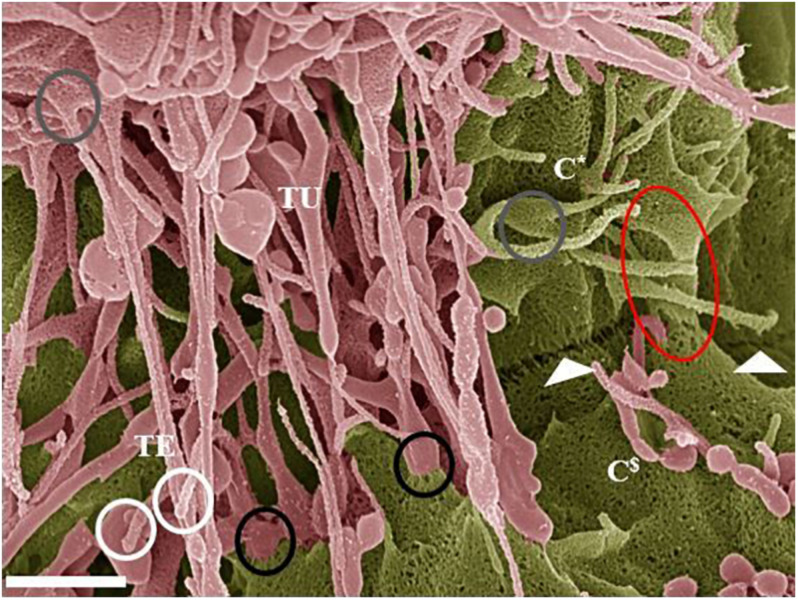
Hemifusion across the PC space between two juxtaposing BECs (Scale bar = 1,000 nm). The HRSEM micrograph depicts juxtapositioning adjacent b.End5 cell membranes and subsequent TJ protein alignment facilitated by TUNT and TENT formation denoted as “**TU**” and “**TE**” within 24 h of monolayer establishment. The occurrence of TENTs and TUNTs together is a clear observation that they have distinctly different functions. The “**white circles**” highlight the extremities of the TENT structures which exhibit bulb-like distal ends. The “**gray circles**” highlight the proximal ends of TENTs, which form tent-like leaflets from the leading edges of the cell membrane. The “**black circles**” highlight the distal ends of TUNTs, which fuse with the surface membrane of the adjacent/target cell, the lower black circle illustrates the short fibers tethering the foot process to the membrane. The “**white arrows**” indicates an additional, novel feature that presents itself throughout the BEC cultures as a partial “stitching” together of adjacent BECs across the PC space (partial fusion). This hemifusion of adjacent BEC cell membranes indicates the site for TJ protein interaction and the “**red circle**” illustrates the beginning of membrane overlapping. The C* denotes cell one and the **C^$^** denotes cell two (the target cell).

The distal ends appear to be continually reabsorbed by the cell’s lateral plasma membranes, inevitably resulting in the shortening of the TENT. The shortening of the TENT appear to have three functions: one, to mechanically pull the membranes of adjacent cells toward each other, and secondly, to align the zones of TJs on the lateral cell walls (see Figures 6, 7), resulting in the hemifusion of adjacent cells. Thirdly, tethering also involves the cell membrane (marginal-folds) being pulled across the PC space by TENTs (Figures 6, 9, 10).

**FIGURE 8:**
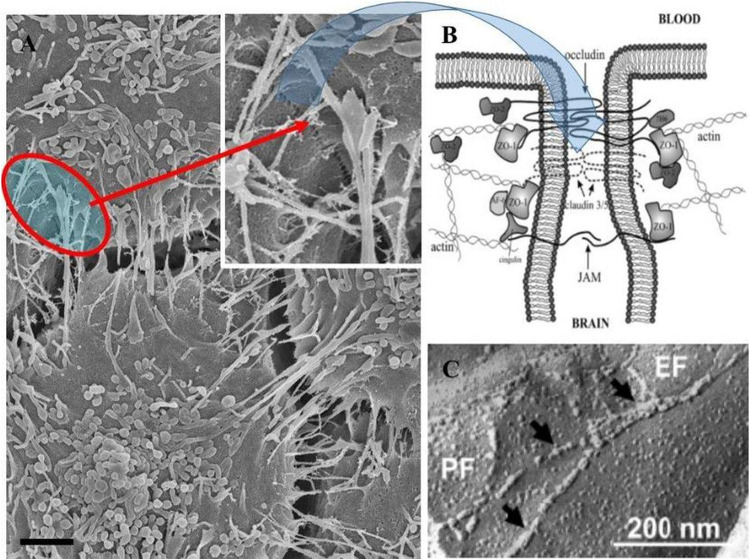
The role of TUNTs in aligning and juxtaposing the adjacent lateral membranes (Scale bar = 2,000 nm). **(A)** Illustrates membrane interaction at the apical domains which are achieved when two juxtaposed BECs are localized in close proximity (see the “red circle”), which shows membranes positioned close. **(B)** Is an annotated diagram of TJ interaction which occurs when adjacent cell membranes are closely position (**B**; Hawkins and Davis, 2005). **(C)** Depicts freeze-fracture electron microscopy of TJ interaction of ECs of the BBB (Haseloff et al., 2015).

**FIGURE 9:**
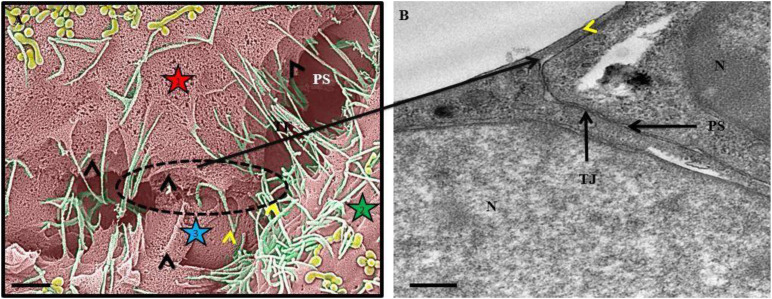
An HRSEM and HRTEM micrograph of tethering nanotubule formation on b.End3 and b.End5 cells. In **(A)** the HRSEM micrograph depicts the formation of membrane tethers which progress into overlapping leaflets between adjacent b.End5 (BECs). The “**black arrowheads**” indicate direct cell-to-cell communication by way of lateral cytoplasmic protrusions of tethering NTs (TENTs). The “**yellow arrowheads**” indicate the overlapping membranous regions generated by TENTs on the lateral borders between adjacent b.End5, BECs. The stars 1, 2, and 3 designate three different cells (Scale bar = 1,000 nm). In **(B)** the HRTEM micrograph depicts an apical membranous region on a b.End3 monolayer, establishing an apparent, continuous membrane surface by the fusion of overlapping membrane regions as indicated by the “**yellow arrowhead**.” “**N**,” denotes the nucleus of the cell, “**PS**,” denotes the PC space and “**TJ**” denotes the region of tight junction localization (Scale bar = 0.5 μm).

**FIGURE 10:**
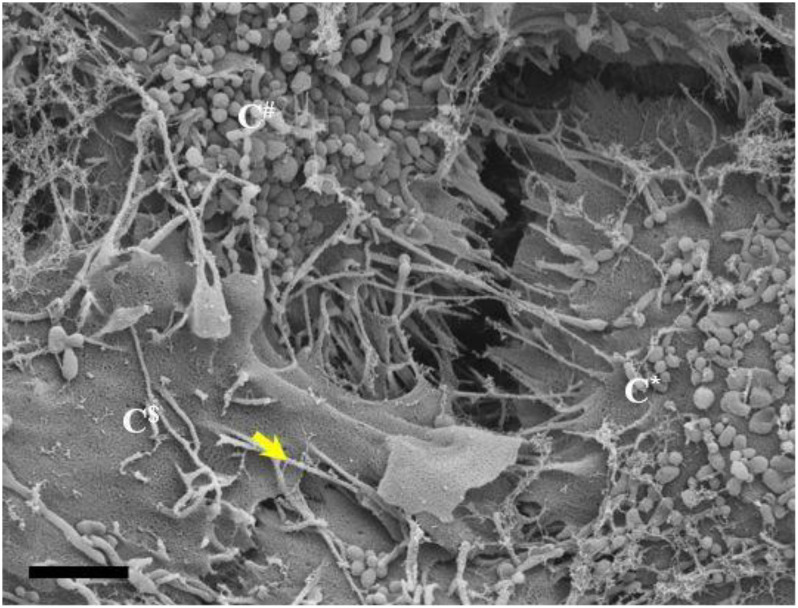
An HRSEM micrograph of two BECs utilizing TENTs to facilitate occlusion of the PC space (Scale bar = 2,000 nm). Short developing TENTs are observed alongside fully extended TENTs, on each of the juxtaposed lateral membrane surfaces of two BECs in close proximity. TENTs are indicated by the “**yellow arrow**.” The micrograph suggests, together with the additional evidence in this paper, that overlapping TENTs may have an important role in BEC membrane continuity. C*, **C^$^**, and **C^#^** denote three different adjacent cells.

The stem of NTs: The stem is represented along the “perforated orange line.” Visually, the TUNT appears larger in diameter and is generated by the fusion of NVs. The TENT is much smaller in diameter, forming tethers, from membranous leading edges of the BEC, as shown by the “perforated black squares,” extending across the PC space. TUNTs and TENTs appear similar in length.

### Hemifusion/Point Cell-to-Cell Interaction

Hemifusion, by definition, refers to the TJ-induced “stitching” together of plasma membranes across the PC space. We hereby document that when b.End5 cells are in close proximity to each other on a monolayer, the plasma membranes of adjacent cells, becomes attached to each other in a “stitched” process across the PC space (Figures 7, 8A). This stitching occurs in the apicolateral domain of the PC space, and each “stitch” has a molecular appearance resembling molecules from opposing lateral membranes “sticking” to each other. It conforms to the theoretical and molecular description for TJs between BECs, the PC freeze-fracture histology of BECs and BEC fluorescence immunocytochemistry postulates of the zone of TJs.

The HRSEM micrograph in Figure 8A illustrates the alignment of the b.End5 cells’ junctional border between the apicolateral domains of two adjacent BECs within the cell monolayer (cell density 1 × 10^6^ cells/insert). The micrograph (Figure 8A) shows that TUNTs and TENTs are candidates to have a role in aligning the PC spaces between adjacent cells, allowing for both mechanical forces to stabilize the PC space, and at the same time, align the adjacent cells to permit interaction between molecular structures (e.g., TJs).

### The Overlapping Apical Membrane

Leading edges of the cell membrane develop into overlapping membrane leaflets. The leaflets are formed by the proximal ends of TENTs. The shaft of the TENT is formed by rope-like, slender tethers that fuse with the adjacent cell’s surface membrane. The TENTS serve to create a series of connections between the apical surfaces of endothelia as a means to cover the zones of apicolateral TJs and further mechanically occlude the PC space.

It is well established in the literature that the apical membranes of BEC, as seen in *in vitro* transmission electron micrographs, form overlapping regions across the PC space (Martins et al., 2013; Haseloff et al., 2015).

TENTs, resembling rope-like tethers, are integrally involved in “pulling across” of the plasma membranes, across the BEC’s PC space, as indicated by the yellow arrows (Figures 9, 10). TENTs have a rigid structure and grow in a targeted direction from the leading edges of the cell membrane across the PC space eventually resulting in the distal ends attaching to the lateral membrane of the adjacent cells. TENT distal ends are not “open-ended” in their architectural make-up, instead, they resemble “closed-ended” nanostructures with “sticky,” distal ends that pull the leading edges of the BEC membrane, generating tent-like overlapping membranous leaflets. The micrograph (Figure 10) depicts the initial stages of cellular communication whereby cells are being drawn toward each other by way of TENTs. The generation of overlapping membrane continuity, in turn, results in an occluded PC space and the protection of the underlying TJ molecular attachments (Figure 10).

### Primary Rat Cardiac Microvascular Endothelial Cell Communication

We postulated that the elaborate intercellular topography we observed in the development of the BEC monolayer was crucial to the formation of a “tight” epithelium where the alignment of TJs were pivotal to the functionality of a “tight” endothelium. The functionality of systemic endothelium, in contrast, is characterized by an absence of TJs, high levels of porosity and permeability. We cultured cardiac capillary endothelium to study *in vitro* monolayer development.

**FIGURE 11:**
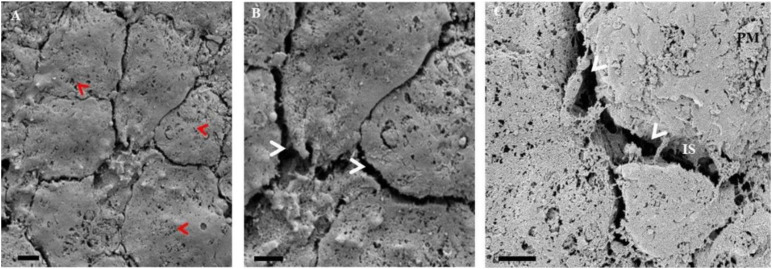
A series of HRSEM micrographs of early stages of the primary rat cardiac microvascular endothelial cell, illustrating the ultrastructural topography of a confluent CMEC monolayer, growing in close proximity. In **(A)** the “red arrows” indicate the pervasive fenestra on the rat CMECs (Scale bar = 2,000 nm). Furthermore, in **(B)** apparent, is the conspicuous absence of TENTs and TUNTs and surface NVs. The “white arrows” indicate the multiple intercellular spaces (IS) devoid of NTs and TJ protein-protein interaction (Scale bar = 2,000 nm). In **(C)** the HRSEM micrograph displays a PC space between CMECs grown in close proximity. The CMEC monolayer is devoid of NVs as indicated by the “white arrows” (Scale bar = 1,000 nm).

After an initial seed (24 h) of the rat CMECs, on a Millicell mixed cellulose esters insert membrane; minimal cell-to-cell interaction is observed. Distinct features of a typical systemic ECs (SEC) display a “cobblestone” appearance when forming a lawn of cells attached to the insert membrane. SECs are distinctly different from the BECs showing a visible reduction in the amount of cell-to-cell communication between adjacent SECs (Plate 11 A). There is a visible absence of ultrastructural NT extracellular projections, in comparison to the complexed PC spaces seen between adjacent BECs (Figures 6–8A). In addition, notable paucity of surface membrane structures and extracellular vesicles are seen on the CMECs, limiting the adhesion of CMECs to both the mixed cellulose esters filter membrane and adjacent SECs growing in close proximity (Plate 11 A). In Plate 11 B, CMECs display clear, uninterrupted PC spaces between CMECs are simple and displays no hemifusion and TJ occlusal interaction, thus demonstrating a typical “leaky”/permeable monolayer expected of systemic capillary endothelia. Furthermore, the CMECs present with multiple fenestra on its membrane surface. Since CMECs do not exhibit exosome formation; little to no NT formation is observed, in contrast to the BECs (Figures 9, 10) of the *in vitro* BBB. Plate 11 C displays reduced intercellular communication, compared to BEC communication. The absence of exosome expression on the cell membrane surface illustrates a very low degree of cell-to-cell communication between CMECs growing in close proximity. A lack of exosome expression subsequently culminated in a lack of direct cell-to-cell contact in the form of TUNT and TENT extracellular protrusions. The paucity of NT formation resulted in the noticeable failure to induce the juxtapositioning of CMEC membranes amounting to no hemifusion of the CMEC apicolateral borders (Figures 11A–C).

## Discussion

Many studies allude to tunneling NTs (TUNTs) being the nexus of biochemical signaling between cells that exhibit extracellular cytoplasmic projections from cell membrane surfaces. This is well-documented in diverse tissues, and crucial for a myriad of physiological processes such as embryogenesis, stem cell differentiation, cell migration and wound healing (Gerdes et al., 2013). Although BECs are central to the establishment of a highly regulated BBB, little is known about the cellular interaction at the level of the PC space. We observed two types of these NTs involved at the PC juncture between adjacent BECs. Using HRSEM to document the sequential interaction at the PC space we postulate the role that these membrane structures play in the formation of a “tight” PC space. In theory, BBB models describing intercellular TJ functionality has been over-simplified. This has been especially conspicuous when analyzing the development of the BBB *in vitro*, using HREM. The micrographical HREM analysis, in this study, documents the snapshot development of the BEC monolayer, as the anatomical basis of the *in vitro* BBB. Nevertheless, these *in vitro* intercellular mechanisms have to correlate closely to the *in vivo* mechanisms of BBB angiogenesis. Direct cell-to-cell communication via ultrastructural interaction of NVs and NTs are novel and special to this study in our pursuit to elucidate, morphologically, the evolution of the BEC into a functional BBB construct.

EC heterogeneity throughout the human body is influenced by its extracellular matrix (ECM), which is promoted by the diverse composition of its basement membrane (BM). Establishing appropriate spatial orientation of a BEC, in culture is paramount to accurately map the progression and orientation of cells during the establishment of the endothelium. The main factor affecting the EC orientation is in selecting a suitable “physiological” base on which BECs can attach themselves *in vitro*. The mixed cellulose esters insert membrane serves as a viable proxy endorsing BM development of the b.End3 and b.End5 cells. In this study, we observe the “secretion” of an amorphous BM which allowed BEC consolidation into a perfectly orientated monolayer, an essential prerequisite for proper expression of domain-specific BBB ultrastructures and protein expression during its development and subsequent angiogenesis (Figure 1). We postulate that this feature is central to the functional orientation of BECs and the TJ enabled sealing of the PC space, a key component of BBB regulation (Fisher and Mentor, 2019).

During growth and development, it is well-reported that cells are inclined to release extracellular vesicles (EVs) of varying sizes (Delmas et al., 2003; Iraci et al., 2016; Datta et al., 2018). We observe, for the first time, EVs sized within an average range of 3–300 nm and, thus, we denote these EVs as NV (refer to Table 1). NVs that have the ability to remain bound to target ECs were reported to transfer proteins anchored within the vesicle membranes, into the plasma membranes of recipient cells according to an early study conducted by Rieu et al. (2000) the literature gives heed to the presence of α4βL-integrin found on reticulocyte exosomes which could bind to the vascular cell adhesion molecule-1 (VCAM-1) on ECs which lead to the cargoing of glycosylphatidylinositol-bound proteins (i.e., acetylcholine) into the plasma membranes of recipient cells, moreover, exosomes are reported to play a functional role in the delivery of prostaglandins to target cells (Samanta et al., 2018). The surface of these membrane-bound, nanosized vesicles are reported to be comprised of saccharide groups, rich in polylactoseamine, α2.6 sialic acid and N-linked glycans (Rieu et al., 2000; Samanta et al., 2018). Similarly, EVs are vehicles for a large number of miRNAs involved in cardiovascular disorders. In the literature, EVs, treated with miR-150 was reported to increase EC migration and miR-126 elevated various types of EVs, promoting re-endothelialization *in vivo*, making EVs an important regulator of angiogenesis and vascular integrity (Samanta et al., 2018) this illuminates the important role for EVs/NVs in a plethora of physiological and pathophysiological functions.

### Relationship Between Exosomes and Nanotubules

By using backscattered secondary electrons, HRSEM allows a resolution of structures approximately 10 nm or less, which permitted us to ascertain sizes of exosomes (Zhou et al., 2007). Upon HRSEM analysis of BECs, grown to 70–80% confluence, enabled the visualization of a myriad of EVs. With reference to Table 1, it is still unclear as to which accepted categorization standards are utilized when classifying the EVs according to function, size and shape. In this study, the micrographical data alludes to potential exosome formation (30–100 nm), however, due to the irregular variation in size from 30 to 300 nm coining these vesicles “exosomes” as the definitive nomenclature utilized becomes imprecise (Figures 2, 4A–C). In terms of its physical properties, the NV exhibits a sticky topographical surface (Figure 2) and attaches to the plasma membrane on its apicolateral surface. Studies conducted by Purushothaman et al. (2016) reports on heparin sulfate material found on exosome membranes which suggests that heparin sulfate acts as a target for fibronectin on the cell surface, alluding to the mechanism whereby these exosomes are “sticky” (Sarrazin et al., 1965; Purushothaman et al., 2016).

Although there have been no reports in the literature regarding the secretion or function of vesicle/exosome secretion from BECs, the phenomenon of exosomes being extruded from cells is supported by work described in LeBleu et al. (2007) and György et al. (2011) and are reported to originate from endocytic vesicles which have been exocytosed onto the cell surface. The contents within a typical exosome depend greatly on the intracellular mechanisms whereby the endosome is reported to be enriched with many bioactive molecules, i.e., proteins, lipids, mRNA and miRNA (Cheng et al., 2017). Cheng et al. (2017), goes on to report that the exosome possesses the ability to traffic paracrine factors, i.e., vascular endothelial growth factor, matrix metalloproteinase, these are proteins vital for angiogenesis within an EC. Also, exosomal studies on mesenchymal stem cells (MSC) reports on its exosomes possessing over 900 proteins. Moreover, exosomes have the capacity to influence the mediation of molecular signaling, by intercellular transferring of information in major biological processes such as cell survival, apoptosis, immune disease and neurological disease (Cheng et al., 2017; LeBleu and Kalluri, 2020).

This study explores an additional class of EVs that form a hybrid vesicle exceeding the size of a typical exosome but does not reach the 1000 nm size as those of the microvesicles. We, therefore, introduce the taxonomic term, “nanovesicle” (NV). The micrographs in Figures 2, 4 suggests that these NVs are of the first nanostructures that appears as BEC populations grow more proximally and its porous nature suggests that it plays an integral role in paracrine communication between cells, a pre-requisite for the transformation of the plasmalemma’s leading edges into NT cross-bridges, therefore, serving as a mediator of cell-to-cell communication. This study proposes that the *de novo* synthesis of the NVs of BECs appears to be triggered by extrinsic paracrine factors released from closely approximated neighboring BECs and is supported by the absence of NVs and NTs on cells growing in sparse populations (Figure 1).

### Primordial TUNTs and TENTs

Actinmyosin bundles have recently been documented in the literature to facilitate adhesion junctions (AJs) within the PC space (Rajakylä et al., 2020). Rajakylä et al. (2020) discriminate between two distinct actin filamentous populations which are separated in semi-confluent epithelial cultures, but upon cell-to-cell contact, morph into indiscriminate cortical actin rings of polarized epithelial cells. It is well reported in the literature that peripheral protuberances of cellular cytoplasm are comprised of actin-myosin bundles, which are contractile and are modulated by Rac1, CDC42 and RhoA activity. These filaments have been documented as running parallel to cell-cell junctions (i.e., TJs, AJs and desmosomes) (Vaezi et al., 2002; Zhang et al., 2005; Gomez et al., 2011; Rajakylä et al., 2020). Conversely, in our study, we observe for the first time, cell membranous NT projections “tunneling” toward target cells, which display distinctly different phenotypical characteristics when compared to the membranous, actin-based leading edges of epithelial cell membranes.

The micrographical data in this study animates two novel NTs which exhibits cellular extensions gravitating toward target PM surfaces of the PC space. It is important to note that NT formation can assume both close-ended and open-ended structures depending on their chronological stage of formation and the process of distal end fusing with the target cell membrane. This expression of the TUNTs are a culmination of cumulative NV vesicles which fuse to form TUNTs. Moreover, these TUNTs extend between two BEC membranes and are a *proviso* required for proper spatial interaction between closely approximated BECs (Figures 3, 6).

### Open-Ended Nanotubes

HRSEM observations show the *de novo* synthesis of the TUNTs induced by the fusion of multiple NVs and the amalgamation of its contents (Figure 4). The TUNTs identified in this study form within a 24–48 h period, and appear bi-directional, ultimately forming “tunneling” tubes between adjacent b.End5 cells (Figures 5–7). Based on the NTs described in this study there are stark disparities between the TUNTs in the current study compared to TUNTs described in the literature.

TUNTs form thin tubular channel connecting adjacent cells across the PC space, permitting cell-to-cell trafficking of biomolecules and organelles (Sáenz-de-santa-maría et al., 2017). The TUNTs in this study appear to facilitate cell membrane alignment and TJ protein interaction at the PC borders of BECs. The membranous interaction induces hemifusion of BEC membranes within the PC domain. The “tunneling” tubes formed by the TUNTs (Figures 4, 6, and 7) result in the juxtapositioning of adjacent BEC membranes suggesting that these NT ultrastructures play an integral role in the transferring of molecular signals which cause BECs to engage in proximal communication to initiate the establishment of a barrier construct. We, therefore, postulate that these fused tubular structures are a distinct category of “tunneling” NTs (Figures 4A–C), and are crucial to the alignment of TJ-zones and the formation of tightly, sealed PC spaces.

NT ultrastructures can give rise to open-ended, tunneling tubes, with varying diameters. According to literature, the diameters of these NTs can range between (50 and 800 nm) (Gerdes et al., 2007) in endothelial progenitor cells and rat cardiac myocytes and (500–2000 nm wide) in neural crest cells (Gerdes et al., 2007; Abounit et al., 2016) with the physical connection being 50–200 nm wide (Rustom et al., 2004). Studies conducted by Gerdes et al. (2013), further support the description of these intercellular structures, as forming transiently, not in contact with the substrate and have been observed as hovering structures within the medium.

**FIGURE 12:**
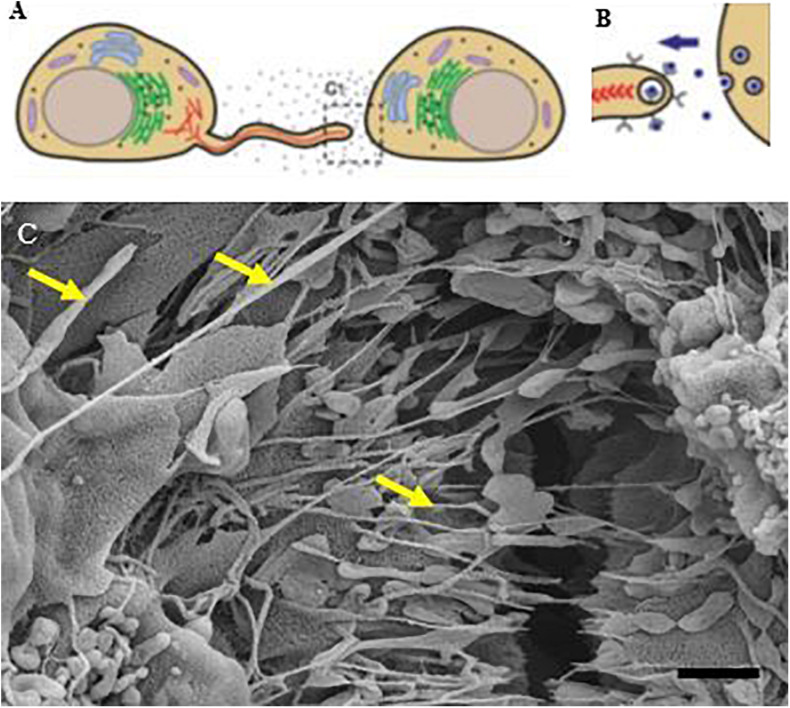
An illustration of basolateral NT progression from membranous filopodia into “closed-ended” structures **(A,B)** (Fulga and Rørth, 2002; Gurke et al., 2008). **(C)** Depicts, morphologically, the formation of TUNTs and TENTs grown on a Millicell substrate and displaying apicolateral projecting nanotubes as seen by the “yellow arrows.” Scale bar = 1,000 nm.

We postulate that the PC ultrastructural NT interactions are directed by a chemogradient which in turn is generated by the paracrine contents of NVs exocytosed onto BEC membranes. We use Figures 12A,B to illustrate the mechanism by which we postulate NTs are attracted to adjacent cells across the paracellular space. The NV gradient is established when BECs are grown on an insert in close proximity (70–80% confluence). Initially, earlier studies, describe extending filopodia as leading edges or motile extensions of the cell membrane border, observed particularly during cell migration, as well as describing filopodial extensions as basolateral cytoplasmic projections. Earlier studies have most likely grown cells on a Petri dish or slides, and not on a surface that allows for correcting cellular orientation and their lateral engagement with each other.

The PC membranous leading edges in this study, however, have direction and are guided by exocytosed, signaling molecules secreted by NVs. The results depict the pulling of plasma membrane folds by NTs across the PC space. We, thus, infer that the sealing off of the PC space is regulated by NT transference of cytoplasmic molecular signals (Figures 6, 7).

### Tethering Nanotubes

In addition to the TUNTs, our study documents the emergence of a second NT, which can be likened to rope-like tethers. In this study, they are denoted as tethering NTs (TENTs). TENTs protrude and pull the cell membrane toward neighboring cells over proximal distances (Figures 9, 10). The extension of the membranous surplus suggests that TENTs have two functions: (i) The primary function, is to mechanically stabilize and align the PC space, so that TJs zones between adjacent cells are aligned, enabling the molecular connection between TJs of adjacent cells, and (ii) it forms a “curtain-like” leaflet over the apical PC space, which fuses with the adjacent (target) cell membrane, forming an overlapping membranous structure, covering the site of TJ and in the case of the BEC, contributes to the occlusion of the PC space, contributing to the integrity of the BBB. Speculation on the purpose of the PC membrane overlap, or membrane fold, suggests that it reduces the shear-stresses of blood flow and assist in the occlusion of the PC space, *as well as* protecting the underlying TJ occlusion zone. We show for the first time how TENTs play an important role in this process.

It is established in the literature that BECs seal their PC spaces by aligning the apicolateral zones so precisely that the TJs on the adjacent cells could align and be closely juxtaposed to each other, for inter-TJ molecular bonding to occur and seal off the PC spaces. Hitherto, it has been unclear as to how these “apicolateral” zones of TJs between two adjacent cells are aligned to allow TJs from adjacent membranes to molecularly bond to each other, creating an impermeable PC seal (Fisher and Mentor, 2019). Thus, both TENT and TUNTs play an integral role in aligning juxtaposed lateral sides of BECs and in the establishing of PC occlusion between the neighboring BECs by approximating adjacent zones of TJs so closely that molecular bonding occurs between TJs of adjacent cells (Figures 6, 7).

The HRTEM experimental model utilized for BECs, grown on inserts, was identical to HRSEM, but was subjected to HRTEM tissue processing. The HRTEM images resemble the features of the *in vivo* TEM micrographs, especially with respect to membrane fold at the PC space and TJ “stitching/interaction beneath the overlapping region (Nitta et al., 2003). These *in vivo* features strongly supports the use of b.End5 cells for use in *in vitro* BBB models.

### Hemifusion

In the literature, TJ (i.e., claudin-5 and occludin) localization is well documented as being restricted to the apicolateral domain of the PC space forming loop-like protein structures that are interconnected with their respective counterparts on an adjacent BEC (Nitta et al., 2003). HRSEM micrographs clearly depict the “stitching together” emulated by TJ protein-protein interaction between adjacent PC apicolateral membranes. TENTs (Figures 7, 8) observably adjusts the alignment of adjacent membranes of BEC growing on the insert membrane. Upon the juxtapositioning of the two membranes, a hemi-junction of the plasmalemma develops upon close contact. We, therefore, propose that TUNTs are requisite for this phenomenon to occur. The TUNT provides the molecular signaling and “cross-talk” between adjacent BECs which is required to facilitate preparing the lateral BEC membranes for a juxtapositioned configuration of the PC space. This direct signaling via TUNTs permits highly specialized TJ molecular zonal interaction, at its apicolateral domains (Figures 6, 7). This study describes, for the first time, the features of an *in vivo* BBB model, in the development of an *in vitro* BBB (DeStefano et al., 2018; Figure 8B). Figure 8B. illustrates theoretical intercellular junctional protein complexes found within the PC space between two adjacent BEC PMs. The proximal location of these adjacent PM leaflets results in the molecular point to point interaction between closely approximated BECs within the apicolateral domain of the PC space. The intercellular interactions in this scenario suggest a strict chronological process that, when unraveled, displays the specific process whereby occlusal apicolateral TJ protein-protein interaction occurs. The partial “stitching together” of TJs are located beneath sealed-off, hemifused intercellular membrane leaflets. TENTs are essential for the formation of overlapping membranes across the PC space and are well described in *in vivo* TEM studies of the brain capillaries. The overlapping membrane across the PC space may play an important role in sealing the PC space and decreasing shear stress at the level of TJs by causing a continuous covering over TJ loci (Figures 9, 10). These findings, therefore, sheds new light on TJ localization and the morphological ultrastructures reinforcing the BBB as a highly regulated and restrictive barrier.

TENTs are “rope-like” nanostructures in this study that attach themselves to the target cells’ plasma membranes, expressing closed-ended, bulb-like projections, with reference to the “white circle” in Figure 7. It could be debated that protruding structures of this nature are likely long-filopodial protuberances, Early in the literature, reports on filopodial extensions morphing into long, slender structures, from the surface of the cell membrane denoted as cytonemes by Gerdes et al. (2007) and Kimura et al. (2012). To date, this particular structure has been observed in a few cell types, namely: T-cells, normal rat kidney (NRK) and neural crest cells (NCs) (Sowinski et al., 2008; Wang et al., 2010; Gerdes et al., 2013). Ramírez-Weber and Kornberg (1999), described filopodial intercellular processes as an established type of cell-to-cell communication.

### Do Systemic Endothelial Cells Display the Same Features?

A comparison of cell-to-cell communication between systemic ECs was essential for elucidating the ultrastructural complexity of BBB development. For the purpose of this study, we have utilized the primary rat cardiac microvascular endothelial cell line (CMEC). We observed, for the first time, the cell-to-cell interaction at high-resolution of CMECs grown on an insert, similarly to b.End3 and b.End5 cells. Our results exhibited a large degree of fenestra on their cell membrane surfaces (Figure 11A). Concurrently, there was a marked absence of NVs on the surface of the CMEC membrane, large PC spaces and little-to-no primordial NT development, compared to BECs (Figures 4, 11). These findings provide us with additional supporting evidence to infer that NTs are pivotal for the establishment of “tight” endothelial barriers and that this level of molecular and cellular anatomical organization is unique to the development of the BBB.

## Conclusion

The novel findings in this study beg the question, “Is BBB integrity and PC occlusion solely orchestrated by TJ interaction?” In this study, we observed the presence of novel ultrastructures: A 3-D representation of a basement membrane (BM), NVs, TUNTs and TENTs. NVs play a significant role in the induction of TUNT formation, by way of paracrine communication. We describe for the first time the novel formation of intercellular NTs that are formed from the fusing of secreted vesicles to form hollow TUNTs connecting adjacent cells, presumably to facilitate the cross-talk between cells to form and align adjacent TJ zones.

The correct BEC alignment is ensured by two important features observed, for the first time, in this study: (i) the extrusion of an amorphous basement membranous structure, formed in the basal domain of BECs during monolayer establishment in culture. The BM ensures that apical and basolateral cellular orientation is achieved. The correct spatial orientation of a BEC allows for the efficient sorting of the PC TJ protein interaction within its respective domains; (ii) the induction of TUNTs bring about the eventual juxtapositioning of apicolateral BEC membranes by forming a scaffolding network of tunneling tubules which facilitates BEC alignment.

Most importantly, closed-ended TENT action results in the overlapping of membranous leaflets across the cellular cleft, establishing an occluded PC space. This study, thus, postulates that intercellular TJ protein-protein interaction is dependent on the formation of dynamic nanoscale networks. Thus, ultrastructural events (i.e., BM establishment, NV and NT formation) strictly govern the molecular underpinnings of BBB integrity and are requisite for the interaction of claudin-5 and occludin. NT-induced juxtapositioning of adjacent BEC membranes sets the precedent for TJ interaction and subsequent hemifusion of its neighboring membranes, which positively contributes to the establishment of a strictly regulated BBB.

## Data Availability Statement

All experimental data collected is archived within the University of the Western Cape (UWC) archives and is available as per UWC data and intellectual property policy guidelines and its associated copyright protection.

## Author Contributions

SM: investigation, methodology, data curation, formal analysis, conceptualization, and writing. DF: supervision, funding acquisition, project administration, conceptualization, and writing. Both authors contributed to the article and approved the submitted version.

## Conflict of Interest

The authors declare that the research was conducted in the absence of any commercial or financial relationships that could be construed as a potential conflict of interest.

## Supplementary Material

Supplementary Figure 1 An HRSEM micrograph of a b.End5 cell expressing NVs and primordial NTs on its cell membrane surface (scale bar = 200 nm).

## Abbreviations

ATCC: American Type Culture Collection
BALB/c: Bagg Albino C57BL/6
BBB: blood-brain barrier
BEC: brain endothelial cells
b.End3: immortalized mouse brain endothelial cells
b.End5: immortalized mouse brain endothelial cells
BM: basement membrane
CMEC: rat cardio microvascular endothelial cells
DMEM:F12: Dulbecco’s Modified Eagles Medium:Hams F12
EC: endothelial cell
ECACC: European Collection of Authenticated Cell Cultures
ECM: Extracellular matrix
Ev: Extracellular vesicle
Flk-1: receptor for vascular endothelial growth factor
HREM: high resolution electron microscopy
HRSEM: high resolution scanning electron microscopy
HRTEM: High resolution transmission electron microscopy
ICAM-1: Intercellular Adhesion Molecule 1
MadCAM-1: mucosal vascular addressin
MECA-32: Panendothelial Cell Antigen Antibody
NT: nanotube
NV: nanovesicle
PC: paracellular
PNVP: perineural vascular plexus
PVP: periventricular plexus
TC: tissue culture
TEM: Transmission electron microscopy
TENT: tethering nanotube
TJ: tight junction
TUNT: Tunneling nanotube.
