# Supplementary figures and images for: High-Resolution Insights Into the in vitro Developing Blood-Brain Barrier: Novel Morphological Features of Endothelial Nanotube Function
Source: Front Neuroanat. 2021 Jun 25;15:661065. doi: 10.3389/fnana.2021.661065 (PMC8267063; doi:10.3389/fnana.2021.661065)

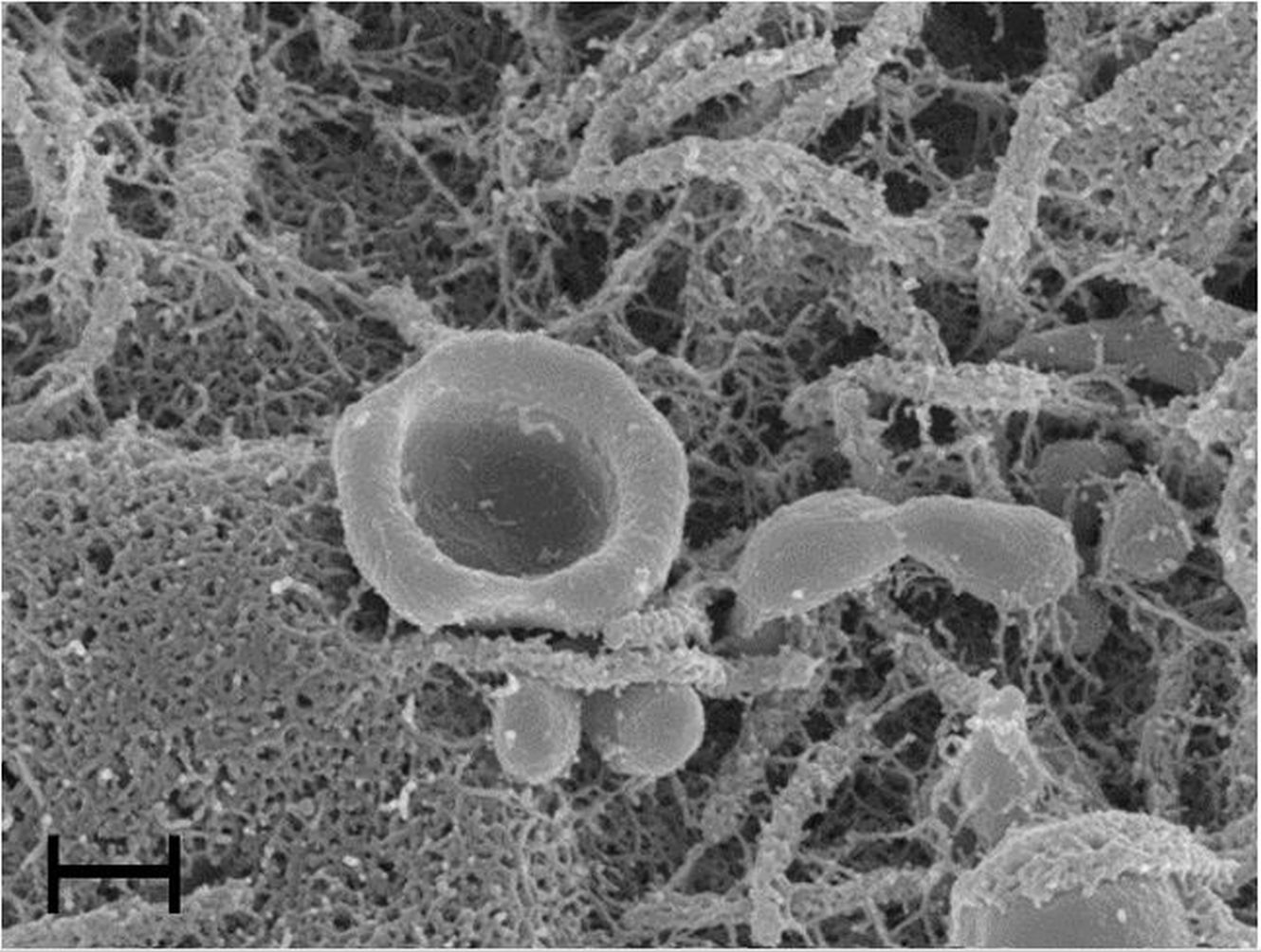

Supplement: Supplementary Figure 1 — An HRSEM micrograph of a b.End5 cell expressing NVs and primordial NTs on its cell membrane surface (scale bar = 200 nm). [file Image_1.jpeg]
